# Cyberbiosecurity Implications for the Laboratory of the Future

**DOI:** 10.3389/fbioe.2019.00182

**Published:** 2019-08-21

**Authors:** J. Craig Reed, Nicolas Dunaway

**Affiliations:** Inspirion Biosciences, Frederick, MD, United States

**Keywords:** biosecurity, cybersecurity, cyberbiosecurity, cyberbiosafety, cyber biorisk management, bioeconomy

## Abstract

Technological innovation has become an integral and inescapable aspect of our daily existence as almost everything of significance in our world now has a cyber (i.e., relating to, or involving computers, computer networks, information technology, and virtual reality) component associated with it. Every facet of our lives is now touched by technology. As such, we're experiencing a digital transformation. Unfortunately, both as individuals and as a society, we're inadequately prepared to embrace the myriad of vulnerabilities presented by cybertechnologies. Unintended cyber vulnerabilities present significant risks to individuals, organizations, governments and economies. Here, we identify current cybersecurity vulnerabilities found in the life science enterprise and discuss the many ways in which these vulnerabilities present risk to laboratory workers in these facilities, the surrounding community and the environment. We also consider the cyberbiosecurity benefits associated with numerous innovations likely to be present in the laboratory of the future. The challenges associated with cyberbiosecurity vulnerabilities are not insurmountable; they simply require thoughtful consideration by equipment designers, software and control systems developers, and by end users. Organizations and the individuals that comprise them must respect, value, and protect their data. End users must train themselves to look at every piece of laboratory equipment and every process from a cyberbiosecurity perspective. With this approach, cyberbiosecurity vulnerabilities can be minimized or eliminated to the benefit of workers, life science organizations, and national security.

## Introduction

Containment laboratories in the United States fall within various economic sectors that comprise the bioeconomy: healthcare and medicine, pharmaceuticals, biotechnology, informatics and agriculture. In 2015, these sectors accounted for $4 trillion or 25% of the US gross domestic product (The National Academies of Sciences Engineering Medicine, 2015). There are over 200,000 biological safety level-2 (BSL-2), high containment (i.e., BSL-3) and maximum containment (i.e., BSL-4) laboratories (labs) in the United States (National Association of County City Health Officials, 2016)^1^^,^^2^^,^^3^^,^^4^^,^^5^^,^^6^^,^^7^^,^^8^^,^^9^. This includes public and private research, biological production, and diagnostic laboratories. These labs are operated by local, state and federal agencies, academic organizations, and for profit and not-for-profit commercial enterprises. A wide variety of public and private sector containment laboratories fall within the US Department of Homeland Security (DHS) classification of Healthcare and Public Health Sector of our national critical infrastructure^10^. This includes Biological Select Agent and Toxin (BSAT) Program labs, state and local public health labs, blood banks, labs associated with medicine and dentistry, and biological production labs that manufacture biological materials for use as vaccines, medical countermeasures and diagnostic reagents (Department of Homeland Security, 2016). DHS describes critical infrastructure as “… the physical and cyber systems and assets that are so vital to the United States that their incapacity or destruction would have a debilitating impact on our physical or economic security or public health or safety. The nation's critical infrastructure provides the essential services that underpin American society^11^.” Information about private sector infrastructure vulnerabilities or data breaches is protected from public release by the Protected Critical Infrastructure Information (PCII) Program if that information is voluntarily shared with the government for the purposes of homeland security^12^. While private sector vulnerabilities are ferreted away, government sector vulnerabilities or data breaches are rarely shared with the public. For example, Title 42. US. Code 262a(h) specifically exempts some information held by the Select Agent program from the Freedom of Information Act^13^. Therefore, while agencies of the federal government have developed awareness of vulnerabilities that exist in these labs, the public, and likely the many individuals who work in these labs, is not apprised of the significant safety and security vulnerabilities present in them^14^^,^^15^. This also means that civilian safety and security solution providers cannot use the information that is known about their vulnerabilities to develop solutions^16^. The cyberbiosecurity risks in containment laboratories, discussed below, represent an additional challenge and make an already complicated situation more complex. In short, the footprint is large, the vulnerabilities are significant, and the consequences are high.

## Current Technology Trends and Their Impact to Today's Laboratories

Disruptive technology trends propel the future and the pace of technological innovation is accelerating. There's no question we've entered a period of digital transformation across all aspects of our existence. “Digital transformation is the change associated with the application of digital technologies in all aspects of human endeavor^17^.” Through this transformation, technology has become a fundamental aspect of our life. Technology now touches everything of significance in our world and everything of significance now has a cyber component. Of importance, our efficiency and productivity are substantially increased when devices and systems are networked and connected to the internet. This efficiency, in turn, accelerates the pace of disruptive innovation.

Despite massive benefit, technology presents significant security vulnerabilities to the life science enterprise. These vulnerabilities must be managed effectively to avoid existential threat to the enterprise, public health, and national security.

Life science labs are in the early stage of transition to the “smart labs” of the future^18^^,^^19^^,^^20^. Most existing labs already possess attributes common to residential properties known as “smart homes.” Smart homes possess networked devices capable of remote monitoring and control such as thermostats, locks, lighting, televisions, and refrigerators. Users can receive auto-notification of service status (i.e., power on/off) as well as physical changes in the environment such as temperature, motion, or sound. This is similar to networked building automation systems (BAS) and energy management software (EMS) commonly found in modern laboratory facilities. These systems provide climate and humidity control and, importantly, control of pressure differentials between work spaces such as administrative corridors and laboratories that operate at varying levels of containment. When networked, building system performance can be controlled remotely and utility consumption and greenhouse gas emissions can be monitored remotely^21^^,^^22^. Some smart systems can schedule recurring preventative maintenance tasks, assign those tasks to specific individuals, and automatically order replacement parts and supplies to maintain stock^23^.

Our smart environments at home and work involve networked hardware and mobile communication devices. They are, therefore, subject to the same cybersecurity vulnerabilities. It's widely recognized that hardware and communication devices such as computers and cell phones possess cybersecurity vulnerabilities and once networked, these vulnerabilities can be exploited by anyone with an internet connection. Poor data security and hardware protection habits in one's personal life combined with a remarkable undervaluation of our personal data may translate to similar behaviors and habits in the work environment. Unfortunately, the general consumer does not routinely utilize recommended and proven cybersecurity practices with their personal electronic devices and data. Consumers tend to use short and simple passwords that can be easily guessed, are reused on multiple devices or across multiple accounts and are rarely changed. They conduct financial transactions across open and unsecure public networks. And they give their personal data away for nothing or almost nothing through enrollment and use of loyalty cards at gas pumps, grocery stores, and pharmacies. These poor personal data security habits translate into similar behaviors and practices in the work environment, thus presenting significant cyberbiosecurity vulnerabilities to the life science enterprise.

Life science businesses and academic laboratories rarely respect the value of or take strong measures to protect information about their work environment because they don't realize its sensitivity or appreciate the magnitude of the safety and security vulnerabilities revealed by such documents. Documents such as floorplans for laboratories and mechanical spaces as well as mechanical/electrical/plumbing schematics reveal the location and magnitude of pathogen storage, research animal housing, mission critical reagents, and network servers. They also reveal the identification and location of video surveillance and intrusion detection devices, facility mechanical systems, critical infrastructure components, inbound utility service connections, outbound liquid waste streams, directional airflow and pressure differentials across rooms. To the knowledgeable adversary, every point of information can reveal significant vulnerabilities of the organization. These same organizations may not have restrictions on employee access to this information. Notably, few organizations recognize they lose control of this information once it's distributed to contractors, vendors or service providers such as those who service equipment, manage renovations, or perform space decontamination.

While some life science enterprises may observe other cybersecurity best practices, life science organizations can be complacent about the security of their networked equipment, generally do not properly value their data and business information, and do not fully recognize the significant security vulnerabilities this information may reveal about their organization^24^^,^^25^^,^^26^. The use of personal devices such as personal laptops and cell phones to access work-related systems results in duplication and redirection of work data streams that introduce additional vulnerabilities and increase the complexity of the cybersecurity challenge for several reasons. First, it requires employers to recognize the necessity to incur the cost associated with either banning the use of personal devices (and issuing company owned devices) or implementing the infrastructure to impose security policies on personal devices that access the organization's networks. Effective cybersecurity policy includes cryptographically strong password usage, use of multifactor authentication, and encryption of data at rest and in transit. While some individuals follow such procedures on their personal devices, most do not. This introduces uncontrolled cyberbiosecurity vulnerabilities to life science enterprise data systems and networked laboratory equipment. Second, personal devices can be used over unsecure public networks—such as in coffee shops or hotel rooms—to access lab systems and data. Without the use of a virtual private network (VPN) or encrypted data, unsecure networks permit other parties to see and intercept transmitted data—a clear vulnerability to any organization. Third, when personal devices are connected to external networks and carried into the lab, they can be used to remove sensitive work data and communicate it to others without detection. Data exfiltration—or data theft—is a perfect example of the insider threat. Fourth, the use of Wi-Fi in a lab or other facility is often a serious vulnerability, and this is exacerbated when allowing personal devices. If a personal device is connected to an organization's internal network and is allowed to broadcast as a Wi-Fi access point, a new point of entry is created for a bad actor. Finally, any mobile device can be lost or stolen. With inadequate security protections, a lost or stolen device can expose the organization's systems and data to intrusion, corruption, and theft. Individuals, businesses, and government agencies are finding that the efficiency and productivity benefits of networking mobile devices, laboratory equipment and facility systems are offset by the crippling security vulnerabilities presented by them. Depending on the size of the organization, remediation of these vulnerabilities can range from a moderately challenging task requiring a single or small number of professionals to a large-scale endeavor requiring a very large team. Regardless, in all cases it requires the organization to implement a risk-based graded approach to information security governance that enables the organization to secure its information, detect loss, and act quickly.

## Biosecurity vs. Cyberbiosecurity

Laboratory biosecurity has been defined as the set of practices and procedures executed at the personal and institutional level necessary to secure and “prevent the loss, theft, misuse, diversion or intentional release of pathogens and toxins” (Meyerson and Reaser, 2002; World Health Organization, 2004). This definition was expanded beyond harmful biological organisms and proteins by Burnette et al. (2013a,b) to include “… products having intrinsic value, such as novel vaccines, biological therapeutics, information technology platforms, synthetic nanoparticles, or organisms, and products having high monetary value or related to biological agents.”

Cyberbiosecurity has been broadly defined by others as “understanding the vulnerabilities to unwanted surveillance, intrusions, and malicious and harmful activities which can occur within or at the interfaces of comingled life and medical sciences, cyber, cyber-physical, supply chain and infrastructure systems, and developing and instituting measures to prevent, protect against, mitigate, investigate, and attribute such threats as it pertains to security, competitiveness, and resilience” (Murch et al., 2018). In this paper we focus our discussion on those aspects of cyberbiosecurity that include all forms of data stored and transmitted through information technology platforms including data streams emanating from networked laboratory equipment, email, electronic documents and files, databases containing sensitive business information, contracts and financial data, raw research data and its analysis, digital inventories of freezer and working stocks, digital genetic and protein sequence, phenotypic and genotypic information about unique recombinant organisms, security access codes, and other intellectual property.

Cyber exploitation of biosecurity vulnerabilities can occur through exfiltration of data by employees or contractors (insiders) or penetration of the organization's networked systems by outsiders. These considerations must be addressed by IT (Information Technology) staff during the collaborative development of a biosecurity program plan (Reed and Sharpe, 2013). Just as the nation's power grid and local utilities are at risk due to the internet accessibility of many individual pieces of networked equipment, so are building automation systems, facility controls and all other networked equipment or communication systems.

Cyber penetration of networked lab equipment and facility controls provides access to the organization's sensitive scientific and business data as well as intellectual property. Aside from denial of service and malware introduction, cyberbiosecurity intrusions and exfiltration of data can result in a cascade of catastrophic reputational and financial outcomes that can challenge the viability of an organization. These outcomes include the destruction, theft, public dissemination of or malicious alteration of electronic genomic and protein sequences, scientific data, intellectual property, and/or security-sensitive facility documents (such as budget documents, program plans, facility floorplans, emergency procedures, continuity of operations plans, etc.). Access to networked laboratory equipment such as freezers, refrigerators and incubators can result in destruction of valuable reagents and microorganisms in long term storage, in use as working stocks, or in active research or experimental use. Networked bench equipment can be turned off and result in lost data and work time. Changes to light, temperature or humidity in animal rooms can result in stress, morbidity or mortality of valuable and expensive research animals. Although we know of no specific events such as these affecting BSAT labs, it is worth noting that only information associated with the loss, theft, release or exposure to Select Agents would be reported to the Select Agent Program —not the destruction of organisms due to a cyberintrusion. The authors are not aware of any requirement forBSL-2 or non-Select Agent BSL-3 labs to report to any authority events such as those described above.

These events can cause irreparable damage to the reputation of individual researchers, principal investigators, specific laboratories, senior leadership of the organization and that of the entire enterprise, institution or federal agency. This, in turn, can erode confidence in the organization by the public as well as current or prospective students, employees, collaborators, sponsors, investors, shareholders and funding agencies. Exploitation of cyberbiosecurity vulnerabilities can be a direct existential threat to the life science enterprise.

## Cyberbiosafety and Cyberbiorisk Management

Cyberbiosecurity is distinguished from cyberbiosafety, which we propose here as a new term for the cyber vulnerabilities associated with networked data systems, laboratory equipment and facility security and engineering controls that may result in environmental contamination or pose a threat to the health of humans, animals and plants including the health of building occupants, the surrounding community, and/or users and consumers of products created by the life science enterprise. Malicious exploitation of cyberbiosafety vulnerabilities include: alteration of electronic genomic sequences to create, enhance or expand infection, host range, pathogenicity or drug resistance of microorganisms (Adam et al., 2011); adjustment of fan speeds in building ventilation systems to alter pressure differentials between administrative and laboratory workspaces which can lead to potential exposure of any building occupant to infectious microorganisms or their toxic products, contamination of the facility, or airborne release of pathogens to the surrounding external environment; and changes to chemical concentration and/or holding time in liquid effluent decontamination systems which can result in premature discharge of infectious, toxic byproducts or genetically altered microorganisms to the municipal waste stream. As with cyberbiosecurity intrusions, the cascade of catastrophic reputational and financial outcomes from cyberbiosafety intrusions represent an existential threat to the life science enterprise and can present a direct immediate threat to the health and safety of building occupants, the public, and the environment.

Although biosecurity, physical security and biosafety are different disciplines, they are synergistic and “… are intimately connected and must be mutually supportive for maximum effectiveness” (Reed and Sharpe, 2013). For any to be fully effective, each must recognize the importance of the other and each must be integrated with the execution of the other. The World Health Organization coined the term biorisk management (World Health Organization, 2006). Biorisk management (BRM) is a management system approach to the identification, elimination and/or mitigation of biosafety and biosecurity risks. We propose here a new term, cyberbiorisk management, as the management system approach to the identification, elimination and/or control of cyberbiosecurity and cyberbiosafety vulnerabilities in the life science enterprise. Detailed discussion of cyberbiosafety and cyberbiorisk management are the focus of a forthcoming publication^27^.

## Current Laboratory Cyberbiosecurity Vulnerabilities and Known Adversarial Events

One of the more mundane but very real risks to data in the life science enterprise is presented by a single piece of ubiquitous administrative equipment, the all-in-one printer/copier/scanner. This networked device stores vast amounts of unencrypted data received from networked computers through print demands in addition to data created through manual copier and scanner functions. This data is not only vulnerable to theft and misappropriation through cyber penetration, it's also readily accessible when the device is physically or remotely serviced, anytime the data storage is removed, and when the current device is replaced with new equipment. Few organizations stop to consider the massive vulnerability this single piece of equipment presents to the security of all forms of business sensitive information created, handled, and stored by the organization including banking and tax documents, contract terms and scope information, intellectual property, personal identification information, HIPAA (Health Insurance Portability and Accountability Act of 1996)^28^ protected information and unpublished research data.

Peccoud et al. (2017) have identified multiple theoretical cyber vulnerabilities associated with networked biomanufacturing process equipment including, supply chain manipulation, alteration of digital genomic sequences, manufacturing process and workflow controls, and the manipulation of process and/or product data. Cyber penetrations that result in alteration of digital genomic or protein sequences could undermine microbial forensics efforts and compromise the ability of the government to distinguish naturally occurring events from deliberate or accidental events. The ability to assign responsibility to malicious actors would be compromised (Reed et al., 2013).

Alteration of processing time and performance of equipment can pose crippling financial and reputational implications due to the loss or destruction of product. On June 27, 2017, the computer networks of the international pharmaceutical company Merck were subject to a global ransomware attack by the NotPetya virus^29^^,^^30^. While this attack was not specifically targeted at Merck's biological production or manufacturing control systems, the attack affected international and domestic operations of the company including biologics production of the pediatric vaccine Garadasil (Human Papillomavirus 9-valent Vaccine, Recombinant)^31^. The attack resulted in at least $135 million dollars in lost sales and $175 million in additional costs during the third quarter of 2017 and forced Merck to borrow $240 million worth of Garadasil from the CDC's Pediatric Vaccine Stockpile^32^^,^^33^. The attack impacted revenue to the same extent during the fourth quarter of 2017, resulting in a total direct cost to Merck of almost $1 billion. NotPetya racked up more than $10 billion in damages worldwide and has been recognized as the most costly cyber attack in history^34^. Despite the direct financial impact to Merck, it's notable that Merck's forward looking statement of risk found in the fourth quarter 2017 8-K Securities and Exchange Commission filing did not identify cyberbiosecurity issues as a potential risk to shareholders^35^.

It's worth noting that future cyber attacks directed specifically at biological production facilities may not only result in the loss or destruction of product, they could potentially result in the creation of potentially harmful products that make their way to end users.

In 2017 at the USENIX security symposium, a group of researchers from the University of Washington presented ground breaking evidence of their ability to encode malware into DNA via a proof-of-concept research project^36^. When the malware-containing DNA was assembled by a gene sequencer, the machine's sequencing software became corrupted. This compromised the computer that controlled the sequencer. Depending upon the networked nature of that computer and the network security protocols in place, this vulnerability could be just the opening an adversary needs to compromise an organization's systems in ways similar to or worse than those described in the paper^37^. This work represents the first demonstration of malicious code insertion into DNA and should be of significant concern to every end user, every gene sequence software developer and every hardware manufacturer.

It's important to emphasize that this work was a proof-of-concept. In phase one of the research, the scientists did not test their theory against a commercially available DNA sequencer/synthesis platform. Instead, the researchers utilized an open source program in which they disabled the security features to create an optimal environment for attack before they introduced the vulnerability. This permitted the researchers to focus their attention solely on the biochemical challenges associated with DNA-based cyber exploitation. Further, the vulnerability introduced by the group was a “buffer overflow.” It is easy to focus on the artificiality of the engineered vulnerability and, perhaps, conclude this somehow invalidates the research. Some might even conclude that this research should have used a novel vulnerability to be useful and that the scenario created by the researchers was so artificial so as to have no intrinsic value. However, this ignores several key points.

First, the use of a buffer overflow as the vulnerability (however artificially engineered), was an interesting choice because buffer overflows have been documented as early as 1972 and are not only one of the oldest known cyber vulnerabilities^38^ but one which often remains unaddressed in modern software releases today^39^. The choice of vulnerability was wise, as it reveals that many software developers over the years have not placed (and still do not place) appropriate priority on security hygiene when engineering their code base. Second, the University of Washington researchers demonstrated this in phase two of their research through their interrogation of poor security hygiene practices in commonly used next-generation sequencing (NGS) and bioinformatics programs. The researchers identified many vulnerabilities, including several buffer overflow vulnerabilities, across different programs.

Simply stated, focusing solely on the vulnerability the researchers chose to exploit may cause some to overlook the critical lessons. Namely, that it is possible (in some cases) to encode malware into DNA, and that many NGS and bioinformatics programs utilize poor security hygiene practices.

Frankly, it's no surprise that life science software developers generally give little to no priority to security hygiene considering that the overall security hygiene in traditional software code is poor. It's imperative this trend be reversed. Manufacturers of NGS, bioinformatics software, and all life science software must consider cyberbiosecurity at the outset of product development, not as an afterthought or with the absence of thought.

Concerns about the cyber vulnerabilities of mobile medical devices captured the public's attention in 2012 when a popular television drama depicted a pacemaker assassination attempt of a fictious political figure^40^. That same year, the US Government Accountability Office identified multiple security vulnerabilities associated with mobile medical devices which are also significant to the life science enterprise (United States Government Accountability Office, 2012), including: unsecure access, unencrypted data transfer, and an inability to update or install security patches or software updates. These vulnerabilities have been exploited by one individual to program an artificial heart to produce a lethal 830 volt shock^41^ and to reprogram an insulin pump to release sufficient insulin to kill its wearer without any warning^42^. In 2013, it was revealed that the unsecured wireless capability of Vice President Dick Cheney's defibrillator was disabled due to the possibility it could be remotely inactivated. The wireless function was intended for software updates to the device^43^.

## Disruptive Technology and Cyber Vulnerabilities in the Lab of the Future—Welcome to the Smart Lab

The Lab of the Future (LotF) will be known as a “smart lab”—a concept simultaneously exhilarating and daunting. “Exhilarating” because the injection of disruptive technology into this workspace will further accelerate the pace and efficiency of innovative research that will, in turn, improve human health, our quality of life and longevity. “Daunting” because the disruptive technology introduces human health risks and security vulnerabilities which must be anticipated, carefully evaluated, and thoughtfully mitigated. Already existing cyber vulnerabilities coupled with the challenges associated with integrating and securing new and disruptive technology may explain why disruptive technology trends have been slower to enter the laboratory workspace than our personal lives. But a number of consumer electronic trends suggest that, ultimately, the LotF will integrate and fully embrace the very same technologies we find ourselves using today at home, as well as those that are just over the horizon.

### Lab of the Future Driven by Virtual Personal Assistants

The portal to the LotF is the voice-driven (or virtual) personal assistant (VPA). According to The Palmer Group, “the world is increasingly mobile and connected^44^.” This same organization identified on-demand services as one of technology's current megatrends. “People are not only willing to access goods and service when they need them, they are getting used to living in a world where their demands are instantly met” (2018 Media & Tech Trend Report). These demands are increasingly met through voice activation services which have now become mainstream because consumers prefer voice activation to typing commands. It's worth noting that speech recognition is 3x faster than typing on smart devices (Ruan et al., 2017).

Siri, Cortana, Google Assistant, and Alexa are some of the most well-known VPAs and are created, respectively, by Apple, Microsoft, Google, and Amazon. Siri, Cortana, and Google Assistant are designed primarily for use on mobile phones or other computer platforms. Alexa is a VPA designed to primarily function inside the Amazon Echo series of smart speaker and video devices. Smart speakers are always-on internet connected devices possessing speakers and omni-directional microphones. Their primary input and output are voice (or voice and video for video enabled devices). Through natural language processing (NLP), location data and access to cloud-stored data, these devices provide audio information directly to users and allow users to access, control and monitor internet-connected products such as thermostats, lighting, security systems, and household appliances. Natural language processing is one of five subdomains of artificial intelligence (AI) and is the technology that enables a computer to both understand and respond in any human language. Natural language processing is what enables a VPA to receive spoken directions and respond with a human voice^45^.

Amazon's smart speakers sold over 22 million units in 2017 and are projected to have a US household adoption rate of 55% by 2022^46^^,^^47^. At this rate, Amazon's smart speakers will become the fastest-adopted consumer electronics device in history^48^. Owners of smart speakers can't do without them−50% use them daily^49^ and more than 30% of owners have more than one device^50^. Park Associates observes, “… voice interface creates a natural gateway to smart home products with consumers desiring to build their ecosystem around voice, thus leading to greater smart home adoptions. [Our] research supports this strong correlation between smart home ownership and adoption of smart speakers with personal assistants^49^.” The ease of voice-based services, combined with the rapid adoption of smart speakers in the consumer market portends an abundance of these devices in the workplace and throughout the life science enterprise.

With such massive numbers for only one popular VPA, it is easy to see how this trend will transfer to the lab. Just as home-based smart speakers are placed throughout the home and used to play music, order pizza, or call a parent; it is really just a matter of time before lab-based smart speakers will be used for similar functions in administrative spaces and laboratories of the scientific enterprise. Smart speakers will be unobtrusively mounted throughout the life science complex in the walls and ceilings of rooms, corridors and laboratories. When flat panel monitors are mounted and networked in the laboratory, conference rooms or huddle rooms, users will request smart speakers to present standard operating procedures, training videos, written documents, and electronic laboratory notebooks (ELNs) on demand. To reduce or eliminate disruption of others in the workplace, smart speakers will be paired with Bluetooth enabled earbuds to enable discrete communication with and receipt of audio content from the networked system of speakers. Individuals will be able to use smart speakers to notify leadership of security and safety emergencies.

Consumer equipment manufacturers wishing to support VPA interactivity currently use the appropriate software development kit (e.g., Alexa Skills Kit, Apple Development for HomeKit, Actions on Google, etc.) to enable their equipment to electronically interface with the NLP capabilities of the VPA. This software is embedded in the software of household smart devices such as lightbulbs, locks, thermostats and refrigerators in “smart homes ^51^^,^^52^^,^^53^.” In the future, software developed by laboratory equipment manufacturers will permit scientists and technicians to use voice commands through smart speakers to control and monitor networked laboratory equipment (i.e., centrifuges, incubators and biosafety cabinets) and data generating equipment (i.e., sequencers and plate readers). The increased use of smart speakers and the expansion of skills will decrease the need for printed documents in the laboratory and accelerate electronic laboratory notebook (ELN) adoption as scientists use smart speakers to dictate select information into e-notebooks, direct the import of data streams from networked bench equipment, and interact with laboratory information management systems. While physical lab notebooks are portable and can be misplaced, lost, damaged or destroyed, ELNs are more secure because they can be encrypted, password protected and stored in the cloud. ELNs that meet regulatory requirements and include the appropriate audit trail and e-signature features may also enhance laboratory quality management, including compliance with Good Laboratory Practices and Good Manufacturing Practices (Kwok, 2018). Software developed by BAS designers will permit facility engineers to remotely monitor and adjust the performance of facility systems. Voice-activated systems and equipment will improve worker productivity and efficiency just as they do in our personal lives. They'll also potentially decrease the likelihood of infection and work surface contamination due to decreased touch in the work space.

### Voice Biometric Authentication as Part of Multimodal Biometric and Multifactor Authentication Improves Security

Current innovations in voice biometric authentication systems (VBAS) combined with smart speaker ease of use will propel smart speaker adoption in the life science workspace. Voice biometric authentication systems such as those currently used by Homeland Security at border crossings^54^ and in the financial, insurance and information technology industries permit the unique identification of individuals based upon their voiceprint^55^^,^^56^. Voiceprints are created from over 100 unique physical and behavioral characteristics that contribute to tone, frequency and cadence of an individual's voice^57^^,^^58^. As the ability of VBAS to rapidly and reliably distinguish individuals increases, it will drive this technology to become an integral component of cyberbiosecurity. When the security enhancement associated with VBAS becomes deployed in the marketplace, many pieces of equipment that are or can be networked in the life science enterprise will benefit from this technology.

Currently, smart speakers and VPAs on the market are user agnostic. While they recognize voice commands, they're unable to distinguish the individuals who issue those commands. Once this limitation is overcome and individual users can be distinguished, integration of VBAS into smart speakers will permit the VPA to distinguish unauthorized users from authorized users and to parse commands based on a user's security authorizations. Once VBAS is integrated, smart speakers will provide life science organizations much greater control of physical security and cybersecurity.

We note that VBAS is not a panacea. It is but one aspect of authentication and by itself is insufficient to provide proper security. To understand why, we must first review the three common categories of authentication. First, is “something you remember or know,” such as your traditional password (Kumar and Farik, 2016). Second, is “something you possess” (Kumar and Farik, 2016). A centuries-old example is a key used to open a lock. Modern examples might include smart cards, software tokens or other hardware devices. Third, is “something you are” (Kumar and Farik, 2016). This includes all biometrics including voiceprint but also includes “…fingerprint, face, iris, retina, gait, palm, and many more…” (Kumar and Farik, 2016).

A fundamental principle in security is the use of multifactor authentication (MFA). Multifactor authentication is the requirement to use two or more forms of authentication to verify the identity of an individual. For example, if an individual wants to log on to a computer or enter a restricted space through a locked door, the individual is required to present something they “have” (such as a smart card), and something they “are” (such as a voiceprint). Alternatively, they could present something they “know” (a password) and something they “are” (a fingerprint). The system does not have to be limited to two factors, it can easily require three or more. In fact, biometric authentication is precisely what many security experts recommend. Because of the limitations of some forms of biometric authentication (such as VBAS) and the ease with which multiple biometric factors (“multimodal biometric authentication”; Kumar and Farik, 2016) can be paired, and the greatly increased security gained by such paring, it is vital VBAS not be discarded by those who only see the risks. Instead, it should be embraced for its ability to enable the life sciences to operate in a radically more efficient environment while remaining safe and secure.

With VBAS using multimodal biometric, and multi-factor authentication, VPAs will not only be able to restrict an individual's physical access to parts of the building, the VPA will also restrict access to networked equipment. It will also support implementation of the organization's IT Security Plan by maintaining access control over business sensitive documents stored on the organization's servers. VPAs with multimodal biometric and multifactor authentication will serve to greatly enhance the security posture of any organization wise enough to employ them.

### Wearables to Monitor Human Performance in High Risk Environments

A relatively undiscussed aspect of containment laboratory operations is physiology, psychology, and human performance monitoring. High containment laboratories (biosafety level-3; BSL-3) and maximum containment laboratories (biosafety level-4; BSL-4) present risks to workers, public health, the environment and national security. Physical medical conditions and mental health issues can impair the ability of individuals to work safely and securely in these environments. Blood sugar imbalances affect dexterity, fine motor skills, vision, balance, clarity of thought, emotional state and executive function^59^^,^^60^. A 2017 report from the US Centers for Disease Control and Prevention (CDC) reveals that 30% of the US population is insulin resistant and displays higher than normal blood glucose levels; they are pre-diabetic. An additional 9.4% of the US population is diabetic^61^. Anxiety disorders—the most common mental illness in the United States—affect 18% of US adults^62^^,^^63^. Additionally, 18% of individuals between the ages of 45–64 were prescribed antidepressants between 2011 and 2014 (Yan, 2017).

We are aware of only two behavioral health screening processes associated with worker access to high and maximum containment laboratories. To assess and monitor the medical and psychological suitability of individuals to work in high and/or maximum containment environments, the U.S. Department of Defense operates a Biosurety Program which establishes a Biological Personnel Reliability Program [BPRP; Department of the Army (2008)]. The BPRP requires medical screening, evaluation, and certification of individuals who have access to BSAT. A medical evaluation is performed to verify candidates are “… free of unstable medical conditions … drug/substance and alcohol abuse and/or dependence …” Disqualifying factors include alcohol-related incidents, alcohol abuse, drug/substance abuse, and “any significant mental or physical medical condition, medication usage, or medical treatment, which may result in … an altered state of consciousness … impaired judgment or concentration.” Individuals are subject to a mental health assessment, as well, and can be disqualified for “… attempted or threatened suicide … extreme moods or mood swings … aggressive/threatening behavior toward other individuals.” Maximum containment laboratory workers at the National Institutes of Health, Bethesda, Maryland are also subject to behavioral health screening (Skvorc and Wilson, 2011). Because activities performed in high and maximum containment laboratories potentially pose unique threats to public health and national security, human performance monitoring through the use of digital health products known as “wearables” may be useful in the future.

The consumer market contains a bevy of wearables including fitness trackers, heartrate monitors and glucose monitors. These devices have become extremely sensitive and permit continuous monitoring of a variety of physiological conditions. For example, the Apple Watch does more than simply monitor heart rate. It can also detect heart arrhythmia with 97% accuracy and hypertension with 82% accuracy^64^. Other devices can monitor blood glucose levels without compromising skin integrity^65^^,^^66^. Approximately 125.5 million wearable devices were sold in 2017 and 240 million are projected to be sold in 2021^67^. When these devices are networked it becomes possible to remotely monitor the vital signs, metabolic status and overall physiological state of individuals^68^^,^^69^. This could prove helpful, for example, for individuals with diagnosed and undiagnosed medical conditions such as hypoglycemia, diabetes, pre-diabetic syndrome, cardiovascular disease or heart arrhythmia. Permission will certainly be required from the individual to be monitored and the resultant data will be subject to protection under the HIPAA. It is reasonable to anticipate that wearable devices such as these will not only be found in the high and maximum containment lab of the future, but also in other work environments, as well. Some will not agree to the value or to the collection of this information and, instead, will view it as an unacceptable invasion of privacy. On the other hand, wearable technologies to monitor human performance are widely used in elite athletic training. However, logical evaluation of the history of data breaches and medical device cyber vulnerability (some of which we have described) could lead one to have concerns. These concerns further reinforce the need for organizations to provide robust, transparent, cyberbiorisk protections to alleviate the vulnerabilities associated with these new technologies. Discussion about the application of wearable technologies in the containment environment are likely to gain acceptance in the future due to the safety and security implications to the individual, environment and society.

### Virtual Reality

Virtual reality (VR) will become a valuable training asset in the lab of the future. Virtual reality replicates or creates an environmental space and is, therefore, perfectly suited for the creation of an exquisitely controlled and focused environment conducive to training. With current technology, trainees can don VR headsets and utilize controllers with basic haptic feedback, to become immersed in a completely safe, risk-free environment, where they will learn by doing. Very soon, more advanced haptic devices such as gloves ^70^^,^^71^^,^^72^ will allow for fully immersive and complex activities. With these training devices available, trainees can be objectively scored on their ability to successfully perform activities like donning/doffing personal protective equipment, preparing a biological safety cabinet for work, or disinfecting a biosafety cabinet following work activities. Training will be self-correcting, through the application of game design principles in non-gaming contexts, defined in Robson et al. (2015) as “gamification,” where trainees increase proficiency through repetitive rounds of practice combined with advancement and reward systems that incentivize progress (Hao and Chuen-Tsai, 2011). Applications that lend themselves to this type of approach include practical testing during biosafety cabinet field certification, training of animal handlers, and BSAT handling activities. With the integration of haptic devices and various hardware components [i.e., gloves, PAPR (Powered Air-Purifying Respirator), bonnet etc.], trainees will be able to sense temperature, vibration, and texture. This will become an essential aspect of training related to animal handling, use of sharps involving animals and activities both delicate and dangerous involving BSAT.

Virtual reality may eliminate the need for trainers or trainees to travel for training events. Instead, trainers will ship VR devices to trainees pre-loaded with instructional software and training content. Trainees will simulate the performance of training tasks at a location, time, frequency, and tempo of their choice. Like a video game, trainees will be able to repeat the simulated events as many times as necessary to move through successively higher levels of achievement to reach their desired level of proficiency. In the end, trainees will demonstrate greater competency in less time through engagement in a fully immersive learning process they can control.

### Artificial Intelligence

According to The Palmer Group, machine learning is one of three megatrends in the field of consumer technology (on demand and autonomy are the other two). “From simple algorithms to complex neural networks, machines are learning to think with us and for us. No matter what you do, there's a thinking machine in your future^73^.”

The implications for VPAs and smart speakers go far beyond touch-free work spaces, voice activation of equipment, document display, and notification of safety/security representatives. The full value of VPAs and smart speakers will be unlocked when scores of organizations network their smart speakers to create a higher order system. At this point, the full digital transformation of the LotF will be underway. Individual pools of data from participating organizations will form a data lake of enough size to permit the application of largescale data analysis, machine learning (ML) algorithms, and artificial intelligence (AI).

AI is the broad science of training a machine to emulate human abilities to perform human tasks^74^ (Turing, 1950; Shannon and McCarthy, 1956; McCarthy et al., 2006; Shubhendu and Vijay, 2013). AI encompasses numerous subfields: machine learning (ML), speech, expert systems, computer vision, robotics, planning/scheduling/optimization, and natural language processing (NLP)^75^. Machine learning applies various forms of data analysis to massive volumes of highly granular and diverse pieces of data to identify broad patterns and draw conclusions (Singh et al., 2016). During the process of data analysis, the massive reservoirs of data are inspected, cleaned, transformed, and modeled to identify useful information, draw conclusions and inform decision-making processes.

Increasingly powerful forms of data analysis require increasing volumes of data (“big data”) and greater computational resources. These analytic processes are called: descriptive, diagnostic, predictive, prescriptive, and cognitive analytics, with cognitive analytics providing insight and outcomes of the greatest value and use. Descriptive analytics draws upon the mining of comprehensive historical and live data to answer the question, “What happened?” (Banerjee et al., 2013). Diagnostic analytics applies cause and effect analyses to identify correlations within data to enable the isolation of confounding information and identification of root cause, thereby answering the question, “Why did it happen?” (Banerjee et al., 2013). Predictive analytics employs algorithms to identify historical patterns and to generate and assess theoretical models to yield predictive forecasts that answer the question, “What could happen?” (Banerjee et al., 2013). Prescriptive analytics is the application of advanced analytical techniques including simulation, optimization and decision modeling to generate best possible recommendations to answer the question, “What should be done?” (Banerjee et al., 2013). Cognitive analytics, in the realm of AI, prompts action or causes something to be done (Gudivada et al., 2016).

The combined application of data analytics, ML and other AI tools to a large and ever-increasing volume of data enables for example, Amazon and Netflix to not only generate personalized recommendations for consumers based upon their individual browsing and purchase behavior, but also to accurately forecast what products and media content will move fastest with any given demographic and to predict when consumers are likely to demand it^76^^,^^77^.

Just as every driverless car in a networked fleet learns from the individual mistakes of every other car in that networked fleet, ML and AI may enable individual laboratories to learn from and avoid the errors, incidents, and accidents that have occurred in any other networked laboratory. If one laboratory notifies the safety office of an incident or an accident, AI will have the ability to analyze the information and enable other laboratories to avoid the issues that led to the incident or the accident. Although massive volumes of data are required for data analytics and AI tools to become effective, once sufficient historical and live laboratory data has been amassed, ML tools will assist institutional security and safety committees in the identification and correction of vulnerabilities associated with administrative controls such as standard operating procedures, animal care and welfare procedures, and security processes. With repeated use, the quality of diagnostic and predictive analytics outcomes and the recommendations produced through prescriptive analytics will be refined, their value will increase, and laboratories will be become more reliant upon them. Ultimately, AI generated recommendations and advice will be provided directly to laboratory staff in real time through the smart speaker or other VPA enabled device to prevent unsafe practices that may cause imminent harm to workers. Connection of video feeds and other security sensors to the system is likely to enable security forces and building occupants to be warned of an imminent security threat.

The application of ML and other AI tools to analyze historical laboratory data, data streams from bench equipment, data from wearables, the online behaviors of users on the organization's computers, downloads, and print demands of business documents, email content, and digitally recorded phone conversations will result in revelatory insights about worker behavior, safety practices, and security procedures. The previous and current actions, behaviors, and physiological conditions associated with the insider threat will be apparent for senior leadership as well as security and safety professionals to recognize. Data analysis of news media—both print and online—as well as insurance case studies, and transcripts of court proceedings and judgements for laboratory-related security and safety claims and awards will be useful in learning from past laboratory accidents and intrusions.

For all the potential helpful benefits that ML and AI tools may bring to the laboratory, there will be well-founded concerns associated with the accessibility and security of the raw data from individual laboratories as well as the aggregated raw data. This information could be used to make inferences about the specific activities, operational details and/or safety and security vulnerabilities associated with a given organization and, potentially, with specific subordinate laboratories.

### Blockchain Technology

Blockchain has been hailed as “… the most important invention since the Internet itself” and is “… an invention like the steam or combustion engine that has the potential to transform the world …^78^.” What makes blockchain powerful is that it works flawlessly and has done so for over a decade as the backbone for cryptocurrencies such as bitcoin^79^. Blockchain can be explained in several ways, some more technical than others. In simple terms, blockchain is a digital audit trail; a shared electronic ledger of all transactions and digital events that is simultaneously secure and verifiable. The “chain” itself is composed of individual “blocks”—or transactions—each of which is attached to the chain in temporal fashion immediately following verification of the current transaction by a majority of users. The blockchain contains a certain and verifiable record of every single transaction ever made in the chain. Accountability is, therefore, 100%. Falsification of or tampering with the transaction is not possible in a blockchain^80^. This is due to the distributed consensus model of the blockchain (as compared to say a traditional centralized database model in which trust in the database requires trust in the entity maintaining it). Because all parties involved with every transaction are recorded in the blockchain, it is impossible for anyone to execute an unrecorded transaction. Therefore, blockchain eliminates the untraceable insider (and outsider) threat. This is a significant distinction from even the most stringent internal security protocols that do not use blockchain. Such systems will always have a weak point, such as a system administrator or executive level security officer who could theoretically bypass security protocols to “cover their tracks.” With the use of blockchain, this is not possible. This should be the most significant aspect of blockchain from an organization's internal cybersecurity and cyberbiosecurity perspectives.

As blockchain technology has become more popular, it has become marketed as a one-stop solution to cyber security challenges. This is misguided at best and can only serve to slow the adoption of this powerful technology. It is important for users to not only understand what blockchain is and what it can do, but just as importantly, what blockchain is not and what it cannot do (by itself). Blockchain is a means to track transactions with utmost confidence. Blockchain can be used to secure private and confidential information such as intellectual property, security plans, or other sensitive data, however blockchain cannot do this by itself. By using blockchain technology as a layer of security (as described above), other security technologies (encryption of data in transit and at rest, multimodal biometric, multi-factor authentication, and many others) can be employed with the confidence that their use will be tracked (by the blockchain) without worry of alteration or manipulation. In other words, by leveraging blockchain technology, the rest of the security systems in place for an organization will be improved and therefore, whatever those security systems are protecting, will likewise see improved protection. It is worth noting this still requires knowledgeable and dutiful security professionals at the helm to manage, monitor, and respond to the data being tracked by the blockchain.

There are myriad financial and non-financial applications for blockchain technology, some of which are already starting to emerge. For example, DHL reported on the potential for blockchain to protect their global logistics^81^, SAP (a German multinational software corporation with over $28 billion of revenue in 2018) is tracking goods from creation to shipment using blockchain to similarly protect their supply chain^82^^,^^83^ and Kodak is using blockchain to digitally protect intellectual property for photographers^84^. In the life sciences, any transaction (digital or physical) that incorporates blockchain technology will gain the benefits of accountability and validation inherent with this technology. Blockchain can be used for chain of custody for BSAT and other high consequence materials, tracking and authentication of laboratory supply chains, authentication of waste management processes, tracking of sensitive documents (digital and physical) and verification of digital genomic and protein sequences. For the benefit of having secure, verifiable and immutable transactional data, blockchain technology can and should be integrated into cyberbiorisk management software solutions of the future.

## Recommendations & Conclusions

The US government has not issued regulations focused on private-sector computer network security, aside from healthcare and financial data laws enacted in 1996 and 1999, even though 90% of US cyberspace infrastructure is owned and operated by private companies and represents the first line of defense in a cyberwar. Instead, the government encourages voluntary improvements to cybersecurity practices saying simply, “The majority of intrusions can be stopped through relatively basic cybersecurity investments that companies can and must make themselves^85^.” To this end, the National Institutes of Standards and Technology has created voluntary computer security guidance to decrease the vulnerability of and increase the resiliency of commercial sector enterprise in the event of a cyber attack (National Institute of Standards Technology, 2018). A 2014 report focused on life science data security conducted by the American Association for the Advancement of Science, the Federal Bureau of Investigation and the United National Interregional Crime and Justice Research Institute came to a similar conclusion, stating: “When evaluating solutions for reducing the vulnerabilities of Big Data in the life sciences, only technical solutions, including access controls and data encryption, exist … Unfortunately, beyond the use of technical solutions and common-sense behavior, institutions and individuals can do very little to address system vulnerabilities” (Berger and Roderick, 2014). While no cybersecurity system is completely impenetrable, this should not preclude individuals or organizations from utilizing proven practices to protect their systems and assets. Doing so makes the task of exploitation more difficult and will likely send an intruder to seek easier targets.

The cyberbiosecurity vulnerabilities of the scientific enterprise identified here and elsewhere can be attributed to several fundamental causes:

- Failure to respect, value and protect the organization's scientific data and business sensitive information;
- The significant security and safety vulnerabilities presented to the organization by sharing or failing to protect this information;
- The increased mobility and interconnectedness of our personal and work-related data and devices;
- Poor cybersecurity practices with personal and work-related data and devices;
- Insufficient emphasis on enterprise-wide cybersecurity and cyberbiosecurity awareness raising, training, competency and compliance monitoring;
- Under estimation of the likelihood of a cyber intrusion;
- Failure to implement a cybersecurity plan that identifies and enforces proven cybersecurity practices including multi-factor authentication and rights management;
- Security vulnerabilities present in networked devices due to poor software design and/or a failure of manufacturers to issue patches for these flaws; and
- An inability or unwillingness of end users to proactively identify, consider and mitigate cyber vulnerabilities associated with networked equipment and systems.

Organizations must do more to acknowledge, mitigate and eliminate the cyber vulnerabilities present in the life science enterprise. Although the impact of a cyber penetration event can destroy an organization's reputation, be massively expensive, and present a threat to public health and national security, significant protection can be achieved with relative ease and small investment. The effective identification, elimination and mitigation of cyberbiosecurity vulnerabilities involves implementation of a management system approach to the application of cybersecurity principles and practices that culminate in the protection, monitoring, and hardening of all aspects of the biosecurity posture of the life science enterprise. To accomplish this, organizations must develop and implement a cybersecurity plan that inspires a culture of conscientious and continual awareness of potential cyberbiosecurity vulnerabilities associated with all communications, business sensitive information, data from networked laboratory devices and facility systems, and physical access to computer terminals. An effective cybersecurity plan will include deployment of a cyberbiosecurity handbook that sensitizes users to the implications of potential vulnerabilities, emphasizes the importance of security vigilance and drives the creation of an organizational culture that appreciates the value of enterprise data and the need to rigorously safeguard it. This does not mean that data and information cannot or should not be shared with collaborators, service contractors or other known parties who have a legitimate and authorized need for it. But it does mean that all staff members must develop a greater awareness of the potential sensitivity of this information and become proactive in its protection.

The cybersecurity plan must be grounded in clear policy stating that all enterprise data will be vigorously protected, limited in distribution, actively monitored for intrusion, theft and leakage, and will never be publicly available online. This means electronic communications, data streams and organizational information are encrypted at rest and in transit to prevent corruption or theft, are subject to secure cloud storage (or secure off-site storage) for redundancy, backup and resiliency to known and emerging threats, and are to be accessed only by known and trusted individuals utilizing cryptographically strong passwords and utilizing a properly implemented multi-factor authentication process. These solutions are commercially available off the shelf.

All organizational information must receive graded security protection through systematic classification (i.e., Public, Project Sensitive, Business Use Only, Restricted, Highly Confidential) and be subject to rigorous access control procedures including rights management to control the ability of individuals to view, edit, download, print and electronically distribute information both internally and externally. Employee access should be limited solely to that information necessary for the performance of their job.

While it's best to not work over unsecure public networks, this isn't always possible, which makes it wise to utilize a virtual private network to protect data communications.

Automated enterprise-wide IT security activities should include automated virus and malware scans for all emails and downloads, training of staff to recognize and report phishing scams, the monitoring of all staff activities on in-house electronic hardware, and monitoring of all data downloads and internet activities on the organization's networks to detect the insider threat.

Distribution of sensitive information to vendors, contractors, and service providers must be subject to non-disclosure agreements and must always be controlled, limited, and encrypted. Prior to distribution, the data owner should require and verify the parties possess appropriate policies, systems, technology and processes to similarly protect the owner's data. Otherwise, these parties represent an uncontrolled vulnerability to the enterprise. Under no circumstances should the parties be allowed to operate the organization's computer terminals, much less be provided direct access to the organization's network or data streams.

All staff members should be trained in the organization's cybersecurity practices with emphasis on the cyberbiosecurity vulnerabilities represented in the organization's data. Routinely, everyone should be assessed for their competence in these practices and actively monitored for compliance. Life science organizations should run anomaly detection software to identify and isolate threats as they emerge. They should also engage in penetration testing to ensure their systems can't be easily accessed from outside.

Future implementation of ML and AI tools to organizational data will present a significant improvement in cyberbiosecurity. These tools will be able to detect and respond to attempted cyber penetrations and thus prevent data theft and corruption from outside the organization. These same tools will enable organization leadership to improve compliance with security policy and practices to protect organizational data from the insider threat.

Aside from the implementation of cybersecurity practices, cybersecurity considerations must become a top priority before the deployment of any technology in the life science space. Scientists and other end users must recognize that every point of electronic interface presents a vulnerability. These individuals must not only train themselves to look at every piece of equipment to recognize and identify these points of vulnerability, they must also scrutinize all data and every process from a cybersecurity standpoint. Only from this vantage point can the user begin to thoroughly and comprehensively address the cyberbiosecurity risks in the life science enterprise. Additionally, hardware and software developers must proactively consider the security vulnerabilities of their products during the development process, not as an afterthought. These developers must make cybersecurity an immediate and fundamental component of their software and product design efforts.

## Conclusions

The U.S. life science enterprise constitutes hundreds of thousands of biological laboratories^86^. Collectively, these laboratories comprise a significant portion of the U.S. gross domestic product (the bioeconomy). These life science laboratories possess cyberbiosecurity vulnerabilities associated with their networked hardware, devices and systems. These vulnerabilities pose an existential threat to individual organizations because exploitation of these vulnerabilities could jeopardize their reputation, the integrity and quality of research data, intellectual property, and biological products. Exploitation of these vulnerabilities could easily compromise the safety of building occupants, public health, the environment and national security.

Cyberbiosecurity vulnerabilities exist in large measure due to inadequate cybersecurity procedures, insufficient respect for the value of the organization's data, a failure to recognize the vulnerabilities revealed within the organization's sensitive business documents, and the failure of individuals to identify and address cybersecurity vulnerabilities associated with networked bench equipment, communication devices and facility systems. Equipment manufacturers and software developers shoulder responsibility, too. They fail to recognize, eliminate or mitigate the cybersecurity vulnerabilities in their products.

The digital transformation of today's laboratories into the smart labs of the future will be ushered in when virtual personal assistants are used to control networked equipment and systems. The application of artificial intelligence to virtual personal assistants networked across many organizations will assist decision making by senior leadership and institutional committees through the identification of cyberbiosecurity vulnerabilities and by providing recommendations for their elimination and/or mitigation. Wearables may be deployed in high risk laboratory environments to monitor human performance. Virtual reality may be widely adopted for training of laboratory staff, especially those performing work with animals and/or BSAT. Application of blockchain technology to create a verifiable and tamperproof record of every transaction made with laboratory data and digital genomic and protein sequences will guarantee the integrity of this information and provide an irrefutable means to authenticate and interrogate all manipulations. Smart labs will increase productivity and accelerate the adoption of additional disruptive technologies. As more networked devices and systems appear in the laboratory, the use of voice biometric authentication as part of multimodal biometric and multifactor authentication will significantly improve cybersecurity throughout life science organizations.

## Author Contributions

JR was the co-originator of cyberbiosafety concept and cyberbiorisk management concept, responsible for structure and content, responsible for considering, incorporating co-author contributions and suggested modifications, and responsible for final version. ND was the co-originator of cyberbiosafety concept and cyberbiorisk management concept, contribution to DNA synthesis hacking, voice biometric authentication, blockchain technology, and review and modifications to ensure overall content quality and readability.

### Conflict of Interest Statement

The authors declare they were employees of Inspirion Biosciences during the period in which this manuscript was prepared and that this manuscript was prepared in the absence of any commercial or financial relationships that could be construed as a potential conflict of interest.

## Footnotes

^1^http://www.gao.gov/assets/660/652308.pdf

^2^https://report.nih.gov/award/index.cfm

^3^https://news.vin.com/VINNews.aspx?articleId=32051

^4^https://www.naics.com/sic-industry-description/?code=8011

^5^https://www.naics.com/sic-industry-description/?code=8062

^6^https://www.naics.com/sic-industry-description/?code=8069

^7^https://www.naics.com/sic-industry-description/?code=8071

^8^https://www.naics.com/sic-industry-description/?code=8092

^9^https://www.naics.com/sic-industry-description/?code=8099

^10^https://www.dhs.gov/cisa/critical-infrastructure-sectors

^11^https://www.dhs.gov/topic/critical-infrastructure-security

^12^https://www.dhs.gov/pcii-program

^13^https://www.govinfo.gov/content/pkg/USCODE-2010-title42/pdf/USCODE-2010-title42-chap6A-subchapII-partF-subpart1-sec262a.pdf

^14^https://www.usatoday.com/story/news/2015/05/28/biolabs-pathogens-location-incidents/26587505/

^15^https://www.usatoday.com/story/news/2015/05/28/labs-fight-for-secrecy/26530719/

^16^https://money.cnn.com/2015/11/30/technology/secret-deals-hacked-companies/index.html

^17^https://www.shellypalmer.com/events/ces-2018/media-tech-trend-report/

^18^https://www.scientific-computing.com/sites/default/files/content/BASL18%20Web.pdf

^19^http://www.digitaljournal.com/tech-and-science/science/four-pillars-of-the-digital-laboratory/article/506737

^20^https://www.rdmag.com/blog/2016/02/digitally-transforming-laboratory-operations

^21^https://www.csemag.com/articles/networked-bas-energy-management-systems/

^22^https://aquicore.com/blog/building-automation-systems-vs-energy-management-software/

^23^https://www.cxalloy.com/home

^24^http://citeseerx.ist.psu.edu/viewdoc/download?doi=10.1.1.123.9572&rep=rep1&type=pdf

^25^https://blog.societyinsurance.com/common-data-threats-and-vulnerabilities/

^26^https://sloanreview.mit.edu/article/your-data-is-worth-more-than-you-think/

^27^Reed, manuscript in preparation.

^28^https://www.govinfo.gov/content/pkg/PLAW-104publ191/html/PLAW-104publ191.htm

^29^https://www.washingtonpost.com/news/the-switch/wp/2017/06/27/pharmaceutical-giant-rocked-by-ransomware-attack/?noredirect=on&utm_term=.4a9d7b51fc4b

^30^https://www.fiercepharma.com/manufacturing/merck-says-its-has-restored-most-its-manufacturing-hit-by-cyber-attack

^31^https://www.apextechservices.com/topics/articles/435235-notpetya-worlds-first-10-billion-malware.htm

^32^https://www.cyberscoop.com/notpetya-ransomware-cost-merck-310-million

^33^https://www.techrepublic.com/article/notpetya-ransomware-outbreak-cost-merck-more-than-300m-per-quarter/

^34^https://www.apextechservices.com/topics/articles/435235-notpetya-worlds-first-10-billion-malware.htm

^35^https://fintel.io/doc/sec/310158/000110465918006007/a18-5152_18k.htm

^36^https://www.usenix.org/conference/usenixsecurity17/technical-sessions/presentation/ney

^37^https://www.usenix.org/system/files/conference/usenixsecurity17/sec17-ney.pdf

^38^http://csrc.nist.gov/publications/history/ande72.pdf

^39^https://securityaffairs.co/wordpress/60507/hacking/skype-buffer-overflow.html

^40^https://www.forbes.com/sites/singularity/2012/12/06/yes-you-can-hack-a-pacemaker-and-other-medical-devices-too/#4bcaa81d6853

^41^https://www.computerworld.com/article/2492453/malware-vulnerabilities/pacemaker-hack-can-deliver-deadly-830-volt-jolt.html

^42^https://www.theregister.co.uk/2011/10/27/fatal_insulin_pump_attack/

^43^https://nakedsecurity.sophos.com/2013/10/22/doctors-disabled-wireless-in-dick-cheneys-pacemaker-to-thwart-hacking/

^44^https://www.shellypalmer.com/events/ces-2018/media-tech-trend-report/

^45^https://towardsdatascience.com/how-amazon-alexa-works-your-guide-to-natural-language-processing-ai-7506004709d3

^46^https://www.forbes.com/sites/gilpress/2017/10/29/22-million-amazon-echo-smart-speakers-to-be-sold-in-2017-driving-us-smart-home-adoption/#2bcd180b481a

^47^https://techcrunch.com/2017/11/08/voice-enabled-smart-speakers-to-reach-55-of-u-s-households-by-2022-says-report/?utm_medium=TCnewsletter

^48^https://adage.com/article/opinion/amazon-alexa-spying/313672/

^49^http://www.parksassociates.com/bento/shop/whitepapers/files/Parks%20Assoc%20-%20Impact%20of%20Voice%20Whitepaper%202017.pdf

^50^https://www.forbes.com/sites/gilpress/2017/10/29/22-million-amazon-echo-smart-speakers-to-be-sold-in-2017-driving-us-smart-home-adoption/#2bcd180b481a

^51^https://developer.amazon.com/alexa-skills-kit

^52^https://developer.apple.com/homekit/

^53^https://developers.google.com/actions/

^54^https://www.globalsecurity.org/security/systems/biometrics-voice.htm

^55^https://biztechmagazine.com/article/2018/11/voiceprint-security-game-changer-banks-and-credit-unions-all-sizes

^56^https://identity.utexas.edu/assets/uploads/publications/Current-Biometric-Adoption-and-Trends.pdf

^57^https://www.theguardian.com/money/2018/sep/22/voice-recognition-is-it-really-as-secure-as-it-sounds

^58^http://www.nuance-media.eu/sites/default/files/pdf/Voice%20biometrics%20FAQ%20Press%20614.pdf

^59^https://www.webmd.com/diabetes/guide/diabetes-hypoglycemia#1-2

^60^https://www.mayoclinic.org/diseases-conditions/diabetes/symptoms-causes/syc-20371444

^61^https://www.cdc.gov/diabetes/data/statistics/statistics-report.html

^62^http://www.mentalhealthamerica.net/issues/state-mental-health-america

^63^https://adaa.org/about-adaa/press-room/facts-statistics

^64^https://techcrunch.com/2017/11/13/the-apple-watch-can-accurately-detect-detect-hypertension-and-sleep-apnea-a-new-study-suggests/?utm_medium=TCnewsletter

^65^http://www.gluco-wise.com/

^66^http://nemauramedical.com/sugarbeat/

^67^https://www.digitaltrends.com/mobile/idc-wearables-maket-2017/

^68^https://www.computer.org/csdl/magazine/mu/2018/01/mmu2018010061/13rRUwInvc3

^69^https://www.computer.org/publications/tech-news/research/wearables-smart-phones-sensing-technologies-mental-illness

^70^https://lmts.epfl.ch/lmts-research/blindpad/dextres-2/

^71^https://haptx.com/

^72^https://www.vrgluv.com/

^73^https://www.shellypalmer.com/events/ces-2018/media-tech-trend-report/

^74^http://jmc.stanford.edu/articles/whatisai/whatisai.pdf

^75^www.cse.scu.edu/~mwang2/ai/AI_subfields.pdf

^76^https://www.predictiveanalyticsworld.com/patimes/how-netflix-uses-big-data-to-drive-success/9693/

^77^https://www.investopedia.com/articles/insights/090716/7-ways-amazon-uses-big-data-stalk-you-amzn.asp

^78^https://scet.berkeley.edu/wp-content/uploads/BlockchainPaper.pdf

^79^https://bitcoin.org/bitcoin.pdf

^80^https://scet.berkeley.edu/wp-content/uploads/BlockchainPaper.pdf

^81^https://newsroom.accenture.com/news/dhl-and-accenture-unlock-the-power-of-blockchain-in-logistics.htm

^82^https://www.nasdaq.com/symbol/sap/financials?query=income-statement

^83^https://www.computerworld.com/article/3298578/sap-pilots-blockchain-based-supply-chain-tracker.html

^84^https://www.digitaltrends.com/photography/kodakone-creates-photo-registry-blockchain-ces2018/

^85^http://archive.defense.gov/home/features/2015/0415_cyber-strategy/final_2015_dod_cyber_strategy_for_web.pdf

^86^Reed, personal data.
